# Characterization of *rag*1 mutant zebrafish leukocytes

**DOI:** 10.1186/1471-2172-10-8

**Published:** 2009-02-03

**Authors:** Lora Petrie-Hanson, Claudia Hohn, Larry Hanson

**Affiliations:** 1 Department of Basic Sciences, College of Veterinary Medicine, Mississippi State University, PO Box 6100, Mississippi State, MS, 39762-6100, USA

## Abstract

**Background:**

Zebrafish may prove to be one of the best vertebrate models for innate immunology. These fish have sophisticated immune components, yet rely heavily on innate immune mechanisms. Thus, the development and characterization of mutant and/or knock out zebrafish are critical to help define immune cell and immune gene functions in the zebrafish model. The use of Severe Combined Immunodeficient (SCID) and *recombination activation gene *1 and 2 mutant mice has allowed the investigation of the specific contribution of innate defenses in many infectious diseases. Similar zebrafish mutants are now being used in biomedical and fish immunology related research. This report describes the leukocyte populations in a unique model, *recombination activation gene 1*^-/- ^mutant zebrafish (*rag*1 mutants).

**Results:**

Differential counts of peripheral blood leukocytes (PBL) showed that *rag*1 mutants had significantly decreased lymphocyte-like cell populations (34.7%) compared to wild-types (70.5%), and significantly increased granulocyte populations (52.7%) compared to wild-types (17.6%). Monocyte/macrophage populations were similar between mutants and wild-types, 12.6% and 11.3%, respectively. Differential leukocyte counts of *rag*1 mutant kidney hematopoietic tissue showed a significantly reduced lymphocyte-like cell population (8%), a significantly increased myelomonocyte population (57%), 34.8% precursor cells, and 0.2% thrombocytes, while wild-type hematopoietic kidney tissue showed 29.4% lymphocytes/lymphocyte-like cells, 36.4% myelomonocytes, 33.8% precursors and 0.5% thrombocytes.

Flow cytometric analyses of kidney hematopoietic tissue revealed three leukocyte populations. Population A was monocytes and granulocytes and comprised 34.7% of the gated cells in *rag*1 mutants and 17.6% in wild-types. Population B consisted of hematopoietic precursors, and comprised 50% of the gated cells for *rag*1 mutants and 53% for wild-types. Population C consisted of lymphocytes and lymphocyte-like cells and comprised 7% of the gated cells in the *rag*1 mutants and 26% in the wild-types.

Reverse transcriptase polymerase chain reaction (RT-PCR) assays demonstrated *rag*1 mutant kidney hematopoietic tissue expressed mRNA encoding Non-specific Cytotoxic cell receptor protein-1 (NCCRP-1) and Natural Killer (NK) cell lysin but lacked T cell receptor (TCR) and immunoglobulin (Ig) transcript expression, while wild-type kidney hematopoietic tissue expressed NCCRP-1, NK lysin, TCR and Ig transcript expression.

**Conclusion:**

Our study demonstrates that in comparison to wild-type zebrafish, *rag*1 mutants have a significantly reduced lymphocyte-like cell population that likely includes Non-specific cytotoxic cells (NCC) and NK cells (and lacks functional T and B lymphocytes), a similar macrophage/monocyte population, and a significantly increased neutrophil population. These zebrafish have comparable leukocyte populations to SCID and *rag *1 and/or 2 mutant mice, that possess macrophages, natural killer cells and neutrophils, but lack T and B lymphocytes. *Rag*1 mutant zebrafish will provide the platform for remarkable investigations in fish and innate immunology, as *rag *1 and 2 mutant mice did for mammalian immunology.

## Background

The zebrafish has many advantages for use in immunological research. Zebrafish can mount innate and adaptive immune responses much like higher vertebrates and their genome demonstrates a high degree of synteny to the human genome [1,2]. Current methods for targeted gene silencing, the availability of many mutant strains and the ease of producing specific mutants make the zebrafish an excellent model for experimental immunology [3]. They are phylogenetically one of the lowest vertebrates and rely more than mammals on innate immune mechanisms, and may prove to be one of the best models for innate immunology. However, the development, propagation and characterization of mutant and/or knock out zebrafish are critical to help define immune cell and immune gene functions in the zebrafish model. Much of the research on inducible components of innate and acquired immunity is based on *in vitro *research utilizing isolated or cultured cells. These types of studies are useful for dissecting specific mechanistic pathways but are artificial, in that unknown cellular interactions that occur in the whole animal are removed from the system. With the production of mutants and specific knock out mice, components of the immune system could be dissected in the whole animal model. The use of SCID and *rag *1 and 2 mutant mice has allowed the investigation of the specific contribution of innate defenses in many infectious diseases. Both mutants have normally functioning macrophages, natural killer cells and neutrophils, but lack T and B lymphocytes [4,5].

Similar mutants are now being used in biomedical and fish immunology related research. Reverse genetics, the ability to inactivate a given gene in an entire animal, is an established technique in zebrafish research [1]. Target-selected mutagenesis was used to identify a series of 15 *rag*1 mutations. One of these was a point mutation that causes a premature stop codon in the catalytic domain of *rag*1 [6]. A functional RAG1 protein is required for V(D)J recombination when generating functional immunoglobulin and T cell receptor (TCR) genes [7]. As in other vertebrates, there is only one functional *rag1 *gene; loss of function at this locus results in a complete block of immunoglobulin gene assembly and, presumably, in immunodeficiency [6]. The aims of our project were to describe leukocyte cell populations of adult *rag1*^-/- ^(mutant) and *rag*1^+/+ ^(wild-type) zebrafish by morphologic and flow cytometric analyses, and to determine the presence or absence of leukocyte-associated transcripts by RT-PCR assays.

## Results

### Differential leukocyte counts

Zebrafish non-erythroid peripheral blood cells included thrombocytes, monocytes/macrophages, granulocytes, lymphocytes and lymphocyte-like cells. Thrombocytes had round nuclei and very scant, faintly eosinophilic cytoplasm that was often irregular. The nuclear chromatin was very dense. Lymphocytes and lymphocyte-like cells included several populations that were round with a high nuclear to cytoplasmic ratio. The cytoplasm stained pale blue and was agranular. Smaller lymphocytes had a very scant cytoplasm. Larger lymphocytes had a more granular cytoplasm. Monocytes were large with a lower nuclear to cytoplasmic ratio. Nuclear shape was round, indented or bilobed, and the cytoplasm was granular, but not frothy. Macrophages were large with a low nuclear to cytoplasmic ratio. The nucleus was irregularly shaped. The cytoplasm was frothy, or vacuolated, and often contained debris, and the margin was often irregular. The granulocyte population was predominately two cell types: a myeloperoxidase (mpo) positive neutrophil and an mpo negative eosinophil. The nuclei of neutrophils were multi-lobed and the cytoplasm stained lilac or pale pink and was not obviously granular by light microscopy. Eosinophils had eccentric nuclei. The cytoplasm stained pale pink and contained larger granules.

**Figure 1:**
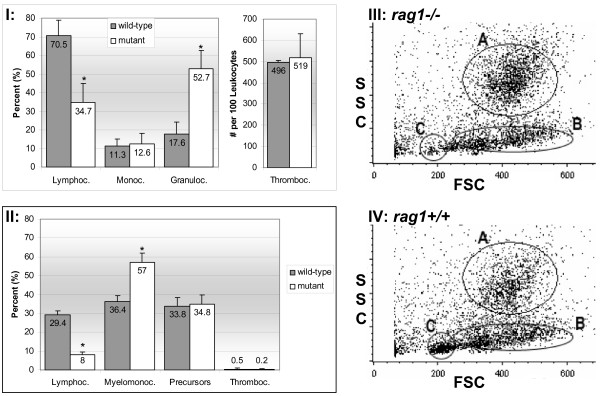
**Mutant and wild-type zebrafish leukocyte differentials and flow cytometry scatter plots**. I and II represent differential counts on peripheral blood smears and kidney hematopoietic tissue smears, respectively. Asterisks indicate significant difference between wild-type and mutant blood cells within the specific population. Average percentage ± standard deviation from 10 replicates is shown (p ≤ 0.05). III and IV show graphs of flow cytometric results on cells from mutant and wild-type kidney tissues, respectively. Graphs represent pooled data from 4 runs on 4 separate mutant and wild-type fish. Forward scatter (FSC) and side scatter (SSC) analyses of whole kidney cell suspension differentiates three distinct cell populations: A-macrophage/monocytes and granulocytes, B-hematopoietic precursor cells and C-lymphocytes and lymphocyte-like cells. Note the reduction in gate C, characteristic of lymphocytes and lymphocyte-like cells, in mutant kidney cell suspensions.

Comparisons of PBL differentials from mutants and wild-types were 34.7% vs. 70.5% lymphocyte-like cells and lymphocytes, 12.6% vs. 11.3% monocytes, and 52.7% vs. 17.6% granulocytes (Figure 1-I). Mutant lymphocyte-like cell populations were significantly less than wild-type lymphocyte/lymphocyte-like cell populations, and granulocyte populations were significantly greater compared to wild-types. Average thrombocyte counts (per 100 leukocytes) were 519 ± 112 (mean ± SD) for mutants and 496 ± 10.6 for wild-types. Comparisons of kidney hematopoietic tissue leukocyte differentials from mutants and wild-types were 8% lymphocyte-like cells vs. 29.4% lymphocytes and lymphocyte-like cells, 57% vs. 36.4% myelomonocytes, 34.8% vs. 33.8% precursors and 0.2% vs. 0.5% thrombocytes (Figure 1-II). Again, mutant lymphocyte-like cell populations were significantly lower than wild-types, and myelomonocyte populations were significantly greater than in wild-types. Cell counts are presented as percentage ± standard deviation.

### Flow cytometry

Examination of mutant and wild-type kidney interstitial cells by Fluoresence Activated Cell Sorter (FACS) analysis demonstrated three main cell populations (Figure 1-III and 1-VI). On the basis of forward scatter (FSC) and side scatter (SSC) properties and location in a FSC vs. SSC plot, these populations were identified as monocytes/granulocytes/myelomonocyte precursors and designated A, hematopoietic precursors designated B, and lymphocytes/lymphocyte-like cells designated C. The FSC threshold was set to exclude erythrocytes. DiOC_6 _and DiOC_5 _staining did not discriminate the zebrafish thrombocyte population from the small lymphocyte population. However, differential counts of kidney hematopoietic tissue indicated thrombocytes were present at less than 1% in this tissue, so discriminating thrombocytes was not necessary. The number of cells with the characteristics of small lymphocytes was significantly reduced in mutant tissue: 7% of gated cells comprised the lymphocyte-like cell population in mutants whereas wild-types had a 26% lymphocyte/lymphocyte-like cell population. We also observed an increased population of monocytes/granulocytes/myelomonocytes in mutants (43%) over wild-types (21%) of the gated cells.

### RT-PCR analyses of mutant and wild-type zebrafish kidney hematopoietic tissues

**Figure 2:**
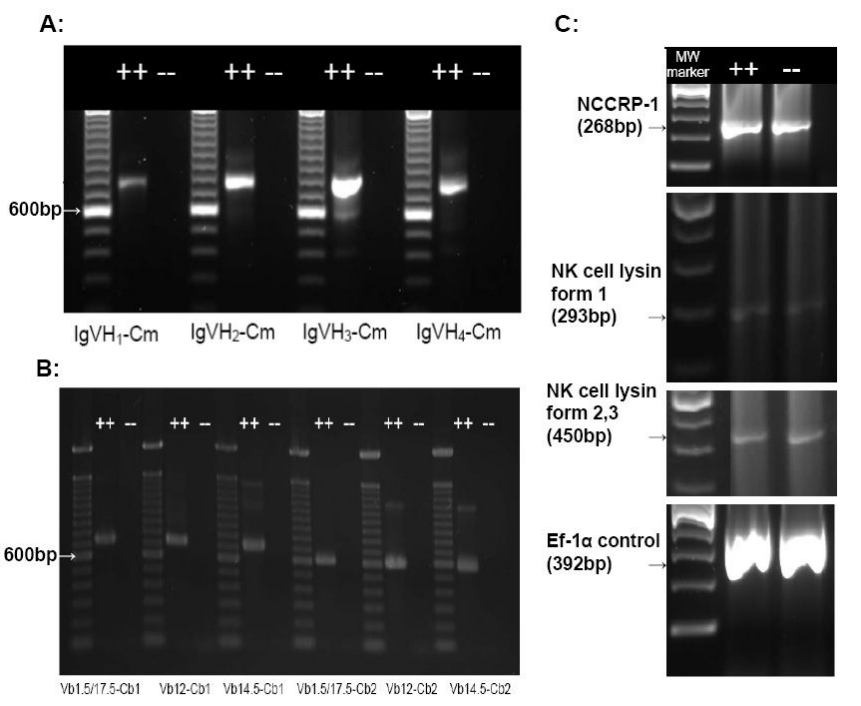
**RT-PCR analyses of mutant and wild-type zebrafish kidney hematopoietic tissue**. Evaluation of expression of Immunoglobulin (Ig) heavy chain gene rearrangements, T-cell receptor (TCR) β chain rearrangements, NK-Lysin, NCC Receptor Protein-1 (NCCRP-1), and transcription elongation factor 1-α (EF1-α, as a positive control) in mutant and wild-type zebrafish by RT-PCR. (A) Nested RT-PCR using primers spanning VDJ-Cm in Ig VH1-VH4 [16]. (B) Nested RT-PCR using primers spanning TCR V(D)J-C_β _[16]. (C) RT-PCR was used to analyze mRNA expression using primers specific for NCCRP-1, NK lysin form 1, NK lysin forms 2 and 3 and EF1-α (primers are listed in Table 1). All RT-PCR assays included no-RT controls and no product was obtained (data not shown).

To determine if kidney hematopoietic tissue included functional B cells, we evaluated mutants for expression of immunoglobulin (Ig) transcripts with heavy-chain gene rearrangements (VH1-Cm, VH2-Cm, VH3-Cm and VH4-Cm). When evaluated using nested RT-PCR, the expected products (Table 1) were obtained for all VH gene rearrangements in the wild-types but no products were obtained from mutants (Figure 2-A). *TCR *genes in zebrafish also undergo V(D)J recombination [8]. To test for functional T cells we evaluated mutant and wild-type RNA for expression of TCR β rearrangements using nested RT-PCR and found expression for these rearrangements in wild-types but no expression in mutants (Figure 2-B). These data indicate that mutant kidney hematopoietic tissue lacks functional T and B cells.

**Table 1:** Oligonucleotide primers used in this study and the expected PCR product size.

**Gene/Fragment**	**Forward Primer**	**Reverse Primer**	**Size (bp)**
TCR V(D) JC complete transcripts from [16]			
Vb1.5/17.5-Cb1 (first round)	aatggacagcttgatagaactgaac	aagatgacaaggccatacagtc	780
Vb1.5/17.5-Cb1 (second round)	tgcttattcaaccgaacagaaacattc	gtccgctcttagcaatggtc	730
Vb12-Cb1 (first round)	cagacaccgtgcttcagtcgag	aagatgacaaggccatacagtc	770
Vb12-Cb1 (second round)	acgtttcatggcagtgttacctg	gtccgctcttagcaatggtc	720
Vb14.5-Cb1 (first round)	gaatccaatgtgacgttaacatgc	aagatgacaaggccatacagtc	750
Vb14.5-Cb1 (second round)	catgatcataaggaccactacag	gtccgctcttagcaatggtc	700
Vb1.5/17.5-Cb2 (first round)	aatggacagcttgatagaactgaac	tgatctccgttgaagaatcggac	550
Vb1.5/17.5-Cb2 (second round)	tgcttattcaaccgaacagaaacattc	gtgcaggtgaatttattgttgggatc	500
Vb12-Cb2 (first round)	cagacaccgtgcttcagtcgag	tgatctccgttgaagaatcggac	550
Vb12-Cb2 (second round)	acgtttcatggcagtgttacctg	gtgcaggtgaatttattgttgggatc	500
Vb14.5-Cb2 (first round)	gaatccaatgtgacgttaacatgc	tgatctccgttgaagaatcggac	550
Vb14.5-Cb2 (second round)	catgatcataaggaccactacag	gtgcaggtgaatttattgttgggatc	500
			
Ig (VDJC) complete transcripts from [16]			
igVH1-Cm (first round)	gatggacgtgttacaatttgge	acatgaaggttgctgatccac	810
igVH1-Cm (second round)	cctcctcagactctgtggtgae	ttgctgatccaccttctaattc	740
igVH2-Cm (first round)	ttgtaacatgaccatgaatatte	acatgaaggttgctgatccac	820
igVH2-Cm (second round)	cgattagatcagtcaccttcte	ttgctgatccaccttctaattc	750
igVH3-Cm (first round)	catgacaatggatattgtgtcce	acatgaaggttgctgatccac	810
igVH3-Cm (second round)	ctctgttggtgtcaaacactge	ttgctgatccaccttctaattc	760
igVH4-Cm (first round)	caagatgaagaatgctctctge	acatgaaggttgctgatccac	810
igVH4-Cm (second round)	tgtcaaagtatggagtcgae	ttgctgatccaccttctaattc	760
			
Zebrafish NK cell receptors (primers designed for this study)			
NK cell lysin 1	ccagctaaagcaaaaccc	ggaaaggtgaaacggaag	293
NK cell lysin 2,3	ttcattcatgttgggcgtgaca	ttgatttcatctggcgttgag	450
NCCR	ggaagctggcagatcacaaca	acggtgtcccaatgcct	268
EF1-α	atggcacggtgacaacatgct	ccacattaccacgacggatg	392

We also evaluated kidney hematopoietic tissue for the expression of mRNA encoding NCCRP-1 and 3 forms of NK lysin using RT-PCR. All RT-PCR assays demonstrated similar amounts of product for mRNAs encoding these NK and NCC specific proteins in mutants and wild-types (Figure 2-C).

## Discussion

Mutant zebrafish had significantly reduced lymphocyte/lymphocyte-like cell populations and significantly increased granulocyte populations. Hematological findings of the wild-type fish are comparable with published values for zebrafish [9]. Published peripheral blood differential results from zebrafish [10] reported 81% lymphocytes, 8% monocytes and 11% granulocytes, and these were comparable to the values we observed: 71% lymphocytes, 11% monocytes and 18% granulocytes. Zebrafish granulopoiesis and myelopoiesis have been well described [11]. The morphologic and functional characterization of macrophages and neutrophils (heterophils) have been further described [12]. Although leukocyte cell counts were significantly different between mutants and wild-types, cell morphologies and staining characteristics were comparable with each other, and other published data referred to above. The thrombocyte populations in mutant and wild-type peripheral blood were statistically the same. Zebrafish thrombocytes have been reported to not be readily distinguishable from lymphocytes [13], but a clear distinction could be made in our study, and was also observed in another study [14].

Kidney hematopoietic tissue was used for flow cytometry, so there was a greater hematopoietic precursor cell population than would be found in peripheral blood. Gate values and cell populations agreed with published flow cytometry data [10,15,16]. Flow cytometry findings corroborate the leukocyte differential counts that demonstrated a significantly reduced lymphocyte/lymphocyte-like cell population in the mutants. Flow cytometric quantifications of brown trout and rainbow trout peripheral blood identified three cell populations that consisted of erythrocytes, lymphocytes plus thrombocytes and neutrophils [17]. DiOC_6 _enhanced side scatter characteristics of carp thrombocytes separated them from lymphocytes [18], and DiOC_6 _and DiOC_5 _were also used to separate avian cell populations [19]. Initial trials with DiOC_6 _were not conclusive in zebrafish. Fortunately, thrombocyte percentages from mutant and wild-type kidney tissues were < 1%, so we did not need to correct for this cell type in our flow cytometry analyses.

Hematopoiesis in zebrafish includes myelopoiesis that forms the myelomonocyte precursor that further differentiates into two myeloid lineages, granulocytes and monocytes [20]. The granulocytes further differentiate into heterophils and eosinophils [21]. The cell populations we identified appeared to directly correlate with the four main cell populations identified by Traver [10]: erythrocytes, lymphocytes, precursors, and myelomonocytes. In flow cytometric analyses of zebrafish peripheral blood, thrombocytes are included in the small lymphocyte fraction. However, as previously stated, we utilized kidney tissue for flow cytometry, and thrombocytes were rarely found in kidney tissue.

The presence of mRNA encoding Ig heavy chain genes and TCR β-chain in wild-type kidney hematopoietic tissue and a lack of their expression in this tissue in mutants indicate that functional T-cells and B-cells are lacking in mutants. Wienholds et al [6] demonstrated a lack of Ig V(D)J recombination using PCR on genomic DNA, but did not evaluate gene expression or TCR gene expression. The RT-PCR findings evaluating transcripts encoding NCCRP-1 and NK lysins suggest that NK-like and NCC cells are present in mutant and wild-type kidney hematopoietic tissue, and these cells likely account for much of the lymphocyte-like cell population observed in the flow cytometry findings. Fish possess both NK-like cells, and NCCs [22]. Zebrafish mutants lacking *rag*1 expression demonstrated a marked reduction or absence of lymphoblasts in the thymus [3], and an increased susceptibility to *Mycobacterium marinum *[23]. *Rag*1 mutant mice also lack functional T and B cells [5], while *Rag *2 mutant mice have functional NK cells [24].

## Conclusion

*Rag1*^t26683 ^mutant zebrafish provide a unique model for investigating innate immune responses because fully functional innate defenses are present without the influence of lymphocytes and presumably lymphocyte associated acquired immune responses. Macrophages are present, as are lymphocyte-like cells, and a significantly expanded granulocyte population. The *rag *1 mutant zebrafish provide the model to elucidate mechanisms of the innate immune system and developmental immunology in fish. Wild-types and mutants were utilized in *Mycobacterium marinum *pathogenesis studies [23]. Additionally, *rag1 *has been shown to be expressed in zebrafish olfactory neurons and *rag1 *negative zebrafish mutants have been used to evaluate the role of *rag1 *in olfactory neuron functions [25]. Future research will provide new tools to direct the level and type of fish immune defenses against pathogens, and *rag*1 mutant zebrafish provide the platform to investigate specific roles of NK-like cells, NCCs, macrophages, neutrophils and eosinophils in fish immune responses.

## Methods

### Mutant and wild-type zebrafish production

Zebrafish used in this study were propagated and reared in the College of Veterinary Medicine's Specific Pathogen Free Fish Hatchery following our Standard Operating Procedures, 2006, CVM-MSU , and housed in a customized zebrafish system [26]. Briefly, adult zebrafish were held in 19 L tanks with charcoal filtered de-chlorinated municipal water at 28°C supplied to each tank at a rate of 0.5 L/min in a flow through, single pass system. Air stones were added to each tank to provide aeration. Water quality parameters were 85.5 mg/L calcium carbonate hardness, 15 mg/L chlorides, 85.5 mg/L bicarbonate alkalinity, and pH 7.2. Adult zebrafish were fed Zeigler™ Adult Zebrafish Diet (Aquatic Habitats™, Apopka, FL) and Golden Pearls Larval Diet (Artemia International LLC, Fairview, TX) brine shrimp (*Artemia*) nauplii twice daily (Aquatic Eco-Systems, Inc., Apopka, FL). Embryos were provided by the Tübingen 2000 Screen Consortium [27]. After rearing to maturity, they were genotyped using a variation of methods previously described [6] and the PCR methods below. Four homozygous *rag *1^-/- ^fish were group spawned to produce the *rag *1^-/- ^mutant zebrafish and twenty *rag *1^+/+ ^fish were group spawned to produce the wild-type zebrafish used in this study. All mutant and wild-type comparisons were made between fish that were between 9 and 12 months old (adults).

### Differential leukocyte counts

Blood was harvested from euthanized adult zebrafish by making a lateral incision just posterior to the dorsal fin in the region of the dorsal aorta [13]. Blood welling up from this incision was collected in a heparinized 10 μl micropipette tip and used to prepare blood smears. Smears were fixed and stained using a Hema-3 Stat Pack (Biochemical Sciences, Inc., Swedesboro, NJ) according to the manufacturer's instructions. Blood cells were viewed on an Olympus BH-1 microscope. Differential leukocyte counts were performed based on morphological appearance, and cells were identified based on previous descriptions of zebrafish or comparative teleosts [12,13,28,29]. Differential counts were made on ten mutants and ten wild-type adult zebrafish. One hundred leukocytes were counted on each slide. Thrombocyte numbers were determined by counting the number of thrombocytes seen while counting 100 leukocytes. Differential leukocyte counts on kidney tissue were done as described above except smears were made from cell suspensions produced from excised kidney tissue. Cell counts are presented as percentage ± standard deviation. Statistical significance was determined using independent Student's t-test. Statistical significance was accepted at p ≤ 0.05. Statistical analyses were performed using SPSS for Windows 15.0 (SPSS Inc., Chicago, IL).

### Flow cytometry

Flow cytometric analyses of mutant and wild-type kidney interstitial cells involved forward scatter (FSC) and side scatter (SSC) determinations on a FACS Calibur (Becton Dickinson). Forward scatter (FSC) represents cell diameter, and side scatter (SSC) represents cell granularity or complexity. The kidney from each of four mutants and four wild-type adult zebrafish were processed. Each kidney was transferred into a centrifuge tube containing 200 μl of phosphate buffered saline (PBS) (0.8% NaCl, 0.02% KCl, 0.02 M PO_4_, pH 7.3) supplemented with 1% fetal bovine serum (FBS), Sigma-Aldrich, St. Louis. MI. Cells were freed from the tissue by repeated pipetting using a P1000 pipettor. The cell suspension was strained, using an additional 200 μl PBS-FBS, through a 40 μm Falcon^® ^nylon cell strainer (Becton Dickinson) to eliminate connective tissue. The cell suspension was kept on ice until analyzed by flow cytometry. DiOC_6 _and DiOC_5 _staining was used to enhance erythrocyte and thrombocyte fluorescent and side scatter properties according to methods for fish and amphibians [18] and birds [19].

### PCR analyses of mutant and wild-type zebrafish kidney hematopoietic tissues

To determine if kidney hematopoietic tissues included functional B or T cells, NK cells or NCCs, we performed multiple PCR reactions on cDNA generated from head kidney tissues of mutant and wild-type zebrafish. To test for functional B cells, we evaluated immunoglobulin heavy chain mRNA expression using nested RT-PCR with primers spanning the V(D)J-Cm region (Table 1 from [16]). To test for functional T cells we utilized published primers spanning V(D)J-C_β _(Table 1 from [16]) in nested RT-PCR to screen mutant and wild-type zebrafish RNA for TCR expression. To determine if the mutant and wild-type kidney hematopoietic tissue included NK-like cells and/or NCC cells, we designed specific primers for three forms of NK lysin and NCCRP-1 (Table 1).

RNA was isolated from zebrafish head kidney tissue using TRI REAGENT^® ^(Molecular Research Center, Inc., Cincinnati, Ohio) according to the manufacturer's protocol. RNA was quantified using the NanoDrop^® ^ND-1000 Spectrometer (NanoDrop Technologies). Prior to RT-PCR, RNA was DNase treated using the RQ1 RNAse-Free DNase kit (Promega Corporation, Madison, WI). For cDNA synthesis from total RNA, the protocol SuperScript™ III First-Strand Synthesis System for RT-PCR (Invitrogen Corporation, Carlsbad, Ca) was followed using oligo (dT)20 as primers. After verification of successful synthesis of first-strand cDNA, 5 μl of cDNA was used for PCR amplification of NK lysin form 1 (NK cell lysin 1) (accession number NM_212741), natural killer cell lysin form 2,3 (NK cell lysin 2,3) (accession number XM690179), natural cytotoxic cell receptor protein (NCCRP-1) (accession number NM_130921), and as positive control elongation factor 1-alpha (EF1-α) (accession number NM_131263.1) (Table 1). The temperature profile used was 35 cycles of 94°C for 30 sec, 58°C for 30 sec and 72 C for 1 min.

## Authors' contributions

LPH conceived of the study, designed the experimental methods for the leukocyte characterizations and drafted the manuscript. CH was involved in all aspects of the study, as it was part of her dissertation project. She maintained the zebrafish colony, performed laboratory experiments and analyses and participated in article revision. LH designed the experimental methods for the genotyping and the RT-PCR and participated in article revision.
